# The Italian Observatory on Food Surplus, Recovery, and Waste: The Development Process and Future Achievements

**DOI:** 10.3389/fnut.2021.787982

**Published:** 2022-01-27

**Authors:** Federica Grant, Laura Rossi

**Affiliations:** Council for Agricultural Research and Economics—Research Centre for Food and Nutrition (CREA Food and Nutrition), Rome, Italy

**Keywords:** food loss, food waste, food donation, legislative framework, measurements, Italy

## Abstract

Food loss and waste (FLW) is an environmental, social, and economic problem. Countries all around the world are looking for efficient strategies to prevent and reduce FLW, as recommended by target 12.3 of the Sustainable Development Goals (SDGs) of the United Nations. The European Union (EU) is strongly committed to helping solve the FLW issue, setting up the Platform on Food Losses and Food Waste, and adopting the Farm to Fork Strategy. Italy has also adopted a consolidated approach toward this issue, in particular through the redistribution of food surplus to those in need, a policy that was instituted with the Gadda Law 166/2016. Importantly, this normative framework also provided for the establishment of the National Observatory on Food Surplus, Recovery, and Waste [Osservatorio sulle Eccedenze, i Recuperi e gli Sprechi Alimentari (OERSA)]. This article describes the creation and development of the OERSA, as the technical entity supporting national FLW policies. One of the first actions taken by the OERSA was that of mapping the FLW initiatives that were being implemented along the entire food supply chain in Italy. This gave the OERSA a solid foundation on which to begin working on two different fronts: (1) Collecting data regarding the primary production sector and at the consumer level and (2) Establishing educational programs and awareness campaigns. The data collected by the OERSA highlight that, although several actors of the Italian supply chain are already conscious of the causes of FLW, new strategies that focus on innovation and cooperation should be encouraged.

## Introduction

Food loss and waste (FLW) prevention and reduction are among the political priorities for national governments and international organizations. It is a subject that is attracting increasing interest, spurring researchers to work on developing prevention, valorization, and management strategies (1–3). The FLW issue is included in the Sustainable Development Goals (SDGs), a universal call for action addressed to all the countries to promote prosperity and protect the planet. The 17 SDGs were adopted by all the United Nations Member States in 2015 as a part of the 2030 Agenda for Sustainable Development, which set out a 15-year plan to achieve these SDGs (4). Each SDG is organized in measurable and monitorable targets. Target 12.3, which aims by 2030 to halve per capita global food waste at retail and consumer levels and reduce food losses along production and supply chains, including postharvest losses, is included in the framework of SDG 12 related to sustainable consumption and production patterns. This means that the entire agri-food chain, from primary production to consumers, must be considered in connection with the FLW prevention strategies.

FLW is a priority also for the European Union (EU) and its member states. In fact, the European Commission (EC) with the Waste Framework Directive (2008/98/EC) (5) defined a “hierarchy” in food waste management to be applied by the EU member states. This strategy, which was further implemented and adapted (1, 3, 6), considers the prevention and reuse of food for human consumption, as the most preferable options, followed by reuse for animal feeding and byproducts, recycling (including composting), and energy recovery, while waste disposal through landfills should be considered as a last resort.

In 2016, the EC established the EU Platform on Food Losses and Food Waste (7), a public and private initiative coordinated by the Directorate-General for Health and Food Safety (DG SANTE), which includes the EU institutions, experts, and stakeholders. The Platform supports all the actors in the definition of food waste prevention measures, the sharing of best practices, and the evaluation of the progress that has been made over the years. In addition, the FLW question is a part of the EU Farm to Fork Strategy released in 2019 (8), a program that aims to achieve sustainable production, processing, distribution, and consumption together with the prevention of FLW.

The Italian normative and cultural background from which the FLW activities were derived is related to the management of food surplus and its redistribution to those living in poverty. The objective of this article is to describe the Italian actions related to FLW and, in particular, the establishment and development of the National Observatory on Food Surplus, Recovery, and Waste [*Osservatorio sulle Eccedenze, i Recuperi e gli Sprechi Alimentari (OERSA*)], a technical entity setup under the scientific guidance of the Council for Agricultural Research and Economics [Consiglio per la Ricerca in agricolture e I'analisi dell'Economia Agraria (CREA)], the national research institution responsible for food, nutrition, and agricultural research (9, 10).

The research questions addressed in this article were: (i) before the establishment of the OERSA, what was the scenario in Italy concerning FLW, in particular regarding the different sectors of the supply chain? (ii) what process was followed in the development of the OERSA and how could this be replicated in other contexts? and (iii) how has the work of the OERSA contributed to tackling the FLW problem in Italy and how can the data collection and methodologies of OERSA be exploited in further research?

This article provides a contribution to the research discussions on food waste action policies and how our experience could be applied in other countries. The description given in this article of the establishment of the OERSA could be used as an example of field work carried out in this area, especially for the identification of key work directives. In addition, the sharing of the main findings of the OERSA, underlining the criticalities related to FLW in Italy, and putting forward different possible solutions to this issue, can provide valuable information based on a concrete experience that could increase the impact of future actions.

The methodology for the preparation of this article was shaped according to the objectives of the work. The OERSA results presented in the following sections were taken from preexisting studies published in scientific papers or scientific-technical reports presented to stakeholders, the main conclusions of which were subsequently elaborated for the purposes of this article. Triangulation was employed throughout the process of data gathering and analysis, comparing bibliographic material, observations, and documents produced within the OERSA framework to obtain information for the future development of the OERSA itself and food waste policies in Italy.

## Italian Policy and Actions on FLW: The National Observatory

### Normative Framework

Italy has adopted a consolidated approach toward food surplus management and redistribution to those in need, codified by a well-defined regulatory process originating in the so-called Good Samaritan Law (155/2003) (11). This law simplified the food donation procedures supporting non-profit organizations in the distribution of food charities. In 2016, an important step forward was taken in the regulatory process with the introduction of the 166/2016 Law (12) that to some extent anticipated the 2030 UN Agenda recommendations. This law provides measures aimed at encouraging food and pharmaceutical surplus redistribution as social solidarity actions through the simplification of bureaucracy, tax deductions, and subsidies either for public or private donors. The objectives of this normative act can be summarized as follows:

- Promoting the recovery and donation of surplus food, primarily to humans, especially to those in need.
- Reducing the environmental impact of FLW through actions that aim to decrease waste and to increase the life cycle of products through reuse and recycling.
- Supporting research activities and increasing consumer and institution awareness, focusing on educating young people.

The Italian board responsible for the FLW activities is the permanent table for combatting waste and promoting food assistance [Food Waste Permanent Table (FWPT)] coordinated by the Ministry of Agricultural, Food, and Forestry Policies. The FWPT organizes activities to reduce food waste at the national level with the aim of disseminating knowledge and sharing data among the key actors in the production system, scientific experts, and society as a whole. The FWPT includes representatives from all the actors of the supply chain (primary sector, manufacturing, industry, retail, and food services), representatives of different Ministries (Health, Environment, and Economic Development), charitable organizations, and Non-governmental Organizations (NGOs) working on food distribution. In this context, the 166/2016 Law established the National Observatory on Food Surplus, Recovery, and Waste as a technical independent entity tasked with collecting and disseminating information and statistics, policy, and best practices related to (i) surpluses along the food supply chain; (ii) food recovery and reuse for human consumption; and (iii) food waste at the household level.

### Development of the National Observatory OERSA

The initial steps in the development of the OERSA consisted of a benchmark assessment of the FLW actions of the members of the FWPT. Semi-structured individual interviews were held with the key representatives of the FWPT who provided documentation, strategy, and policy documents where available. For the semi-structured interviews, a broad framework of open questions was used, allowing the dialog to be shaped and adjusted to follow the line of the most informative topic areas emerging from the responses of the interviewee. Interviews were then matched with the documentation provided and triangulation was carried out, comparing interview material, observation, and documents from the various sources. Based on this information, a map of the FLW initiatives was produced to identify potentialities and barriers for a further reduction in FLW, the actors and stakeholders involved along the food chain, and overall to gain an insight into the stage of development of the national FLW polices. An evaluation of the level of initiatives was then done in terms of projects and best practices including the degree of involvement of local communities, the availability of impact indicators and monitoring data, and the added value of the initiative. Critical issues, improvement practices already implemented, strategies, and overall good practices for the prevention of food surpluses and waste were collected and compared for monitoring purposes.

The level of integration of the results collected from different stakeholders was described in terms of performance and output of ongoing actions. The strengths and weaknesses of the different approaches, common intervention, and entry points as well as gaps to be filled were identified. The results of this assessment are shown in the first-year report of the OERSA (13) and summarize in Table 1.

**Table 1:** Assessment of actions and projects related to the food loss and waste in Italy as a benchmark of the OERSA development.

**Sector**	**Main findings**
Primary sector	The Regional Agency for the supply in agriculture (AGREA) set up a platform which collects data and information on the recovery of agricultural products. Food recovered for human distribution varies from 6,881 tons to 27,671 per year. Peaches, clementine, melons, watermelons, onions, nectarines, kiwis, plums, pears and apples were the foods items collected most frequently.
Wholesale sector	The Italian network of markets (*Italmercati*) quantified the amount of fruit and vegetables products recovered from the market and donated through charities that accounted for 4,000 tons in 2016, corresponding to a monetary value of 8 million euros. Recovery actions were concentrated in northern Italian regions with the markets in Milan and Verona covering 70% of the donations.
Retail sector	The National Consumers' Cooperative Organization (ANCC-COOP) during the period 2013-2016 found that between 1.2 and 1.4% of food in their supermarkets became surplus. The food surplus redistribution accounted for 6,000 tons, 80% of which consisted of fresh and perishable products.
Food service sector	The National Association of Catering Companies that has an Observatory on catering and nutrition (ORICOM-ANGEM) in 2015 performed a survey in a sample of school canteens in Northern Italy aimed at making a qualitative, quantitative and economic estimation of food waste. Meal leftovers were 12.5% of the food prepared, especially side dishes. For each meal, the waste was estimated at a monetary value of 0.18 euro.
Life project, an integrated approach, from the industry to the consumer	This project undertook an in-depth analysis across different stages of the food supply chain, focusing on food industries, large-scale distribution and consumers. A survey carried out in 2018 among 40 companies highlighted that 60% of food surplus consisted of meat and cured meat, pasta and bakery products, frozen and “IV gamma” products. In the retail sector, fresh products such as dairy products, fruit, vegetables, meat, and fish had greater excess than processed food. 90% of the food surplus was disposed of into landfill and only 10% was donated to non-profit organizations. The consumer survey highlighted that the categories of food that created the highest amount of waste were fruit, vegetables and prepared meals, with bread proving to be the food that was thrown away the most often. The main causes of waste were the presence of mold, passing the best before date and changes in sensory characteristics. Nevertheless, 70% of the sample reported knowing the difference between use-by and best before date.
Recovery sector/ Charity organizations	The information platform of the Banco Alimentare organization represents a valuable information source in terms of the accuracy and the large number of data collected, as well as for the coverage of the national territory. The database analysis highlighted that, as a result of Law 166/2016, from 2016 to 2017 the quantity of food recovered increased of 2,100 tons. The collection of this data makes it possible to establish which sectors are keener on surplus donation respect to those in which actions to promote donations are needed.

The meetings with the key informants of the FWPT showed their willingness to share knowledge and data and at the same time allowed the OERSA members to identify in which sector further studies and interventions needed to be undertaken. As a result, the OERSA developed two directives as a guide for the actions to be implemented:

#### Directive Action 1: To Fill Information Gaps

- Collecting primary sector data and information through the Agencies for Agricultural Supply, at the national and regional levels, carrying out an exploratory survey on the primary sector companies.
- Collecting information at distribution and consumer level.
- Carrying out an exploratory survey on the food service sector.
- Carrying out qualitative and quantitative surveys with consumers.

#### Directive Action 2: To Improve Policy and Intervention Actions

Carrying out educational programs aimed at increasing the awareness of food chain operators and consumers of the importance of preventive actions to be established, alongside counteractions for food surplus redistribution and recovery. Two other actions aimed at reducing or preventing FLW during redistribution were identified: the optimization of the use of instruments that guarantee the correct storage of fresh and processed foods and the improvement of the logistic structure of charitable organizations.

## Actionable Recommendations in Italy

### Main Results of the OERSA Data Collection

In accordance with these two action directives, during their first 2 years of activity, the OERSA carried out an assessment on household food waste and an evaluation of food surplus, losses, and waste in the primary sector (Directive action 1). These activities were considered as a valuable tool in drawing up policy actions (Directive action 2). During coronavirus disease 2019 (COVID-19) pandemic, considering the exceptionality of the situation, the OERSA undertook a special assessment to monitor the food habits of Italian consumers and to understand their attitude toward food waste during the period of lockdown between March and April 2020. The results of the data collection were collected in 3 reports (13–15) and 3 peer-review publications (16–18).

The OERSA household food waste measurement was the first assessment done at the national level with a representative sample of the Italian households. The data were collected using the methodology developed by van Herpen et al. (19) that allowed a comparison with other European countries (20). The survey involved a representative sample of 1,142 Italian households. The Italian families wasted, on average, 370 g of food per week—a quantity that is in line with that of the Netherlands (365 g/week) and progressively different from Germany (425 g/week), Hungary (464 g/week), and Spain (534 g/week). Perishable products, such as fresh fruit and vegetables, bread, and non-alcoholic drinks, were those with the highest level of waste. Unused (43.2%) or partly used (30.3%) products were the categories thrown away most frequently. There was a significant association between household food waste and preventive practices and the ability to reduce the amount of food that had to be thrown away. The detailed results of this study were released through an OERSA report (in Italian) (13) and a scientific paper (16). Data on household food waste were further linked and duly elaborated with the data on food product purchases in supermarkets and large-scale retailers as the amount of food bought and its economic value (18). This study aims to evaluate the weight and monetary values of food waste among a sample of the Italian families, which reported a total amount of 399 kg of food waste per week (4.4% of the weight of the overall food purchased), corresponding to a monetary value of €1,052 (3.8% of the overall food expenditure). Clustering the food groups according to waste quantity, typology, and monetary value, it was possible to show that price has a role in the generation of food waste, as the lower the unitary cost, the higher the quantity of waste. Consequently, foods with high unitary costs were those less likely to be thrown away.

The OERSA studies for the second-year report focused on agricultural production (14). Two assessments were carried out for this purpose. The first was an analysis performed on preexisting data on agricultural surpluses. The results showed that in 2017, about 3% of fruit and vegetables were left unharvested. Losses were greater in the area of vegetable cultivation (4%) than fruit cultivation (2.6%). Data concerning unharvested fruits and vegetables vary significantly for every product and can fluctuate considerably depending on the harvest every year. Focusing on single crops was helpful to identify the most problematic production with a high risk of market imbalances. The establishment of the Producer Organization (PO) system that brings together farming companies and cooperatives created competitive advantages in preventing criticalities generated by food surpluses and, therefore, also food waste—in the case that the surpluses were distributed to the poor. In 2017, 32,237 tons (0.3% of production) of fruits and vegetables were collected as surplus, the majority of which (82%) were subsequently distributed for consumption by those in need.

Considering the importance of the POs in the redistribution system, a survey of the 17 POs was then carried out. The four fruit supply chains with the highest market excess (apples, kiwis, peaches, and plums) were analyzed, to evaluate the percentage of the production, main causes, and possible interventions regarding the food surplus generation. The tendency to generate food surplus was higher for plums, lower for kiwis, and variable for apples and peaches. The POs declared that the primary reasons that led to food surplus were changes in weather conditions (86.1%), the presence of parasites or phyto-pathologies (63.9%), alongside problems related to the shape or the size of the products (61.1%). The main food surplus destination was human redistribution (64.9%), followed by distillation (40.5%), composting (35.1%), and the processing industry (18.9%). These figures demonstrated the efficiency of the POs system in preventing food waste by the redistribution of surpluses to the poor.

The OERSA special issue on COVID-19 (15) aimed to evaluate how the Italian habits changed during the lockdown period, the determinants of the changes, and the effect on food waste prevention. The results showed that the lockdown in Italy improved the overall quality of diet, but had negative effects both on the quantity of food consumed and on the levels of physical activity. This situation led to a general increase in body weight and to an exacerbation of the sedentary lifestyle that were already common in Italy. In addition, it highlighted that nearly 80% of respondents were sensitive to food waste, signaling the increased awareness of the Italian consumers of the negative consequences of throwing away food (17).

### OERSA Practical Outcomes

As mentioned above, the data collected by the OERSA were aimed to provide a benchmark assessment as a reference for further monitoring as well as to improve policy and intervention actions. The first step in the development of the recommendations was centered at a consumer level, which was also the main food chain sector analyzed by the OERSA. The results of the assessment (13–18) allowed the OERSA to understand which behaviors are more closely connected with the wasting of food. The high prevalence of fresh products that were thrown away is in line with the perception of not having a clear idea of how to store these kinds of food correctly. In addition to this, wasting unused and partly used food rather than leftovers points to the fact that the consumers may have had difficulties in understanding whether a product was still edible, but also in having adequate equipment or space to store the food. Moreover, price plays a role in generating waste, considering that the Italian consumers tended to throw away less costly foods that were bought in higher quantities.

The OERSA awareness campaigns were based on the observatory results which were also confirmed by other studies carried out in different samples (21–23) and in different settings (24–27).

Several different tools were used for drawing up the recommendations, specifically:

- Website: The OERSA official website (to go live soon) was setup describing in detail how the OERSA was established, its mission, and its main tasks. The website is considered the best communication tool between the OERSA and those who are interested in the subject of FLW. In addition, on the CREA—food and nutrition center website—there is a section^1^ that briefly describes the work of the OERSA, where documents and papers are published.
- Weekly updates on social networks: The OERSA created a profile on various social networks where scientific content is published. The Facebook^2^ and Instagram^3^ profiles are in Italian, while its Twitter^4^ account is in English and had around 250 followers at the time of publication. The idea behind the use of social networks was to involve several population groups and to reach an international public. In fact, even with summarized and simplified content, the social network is likely to permit a more immediate diffusion of information than the official websites.
- The Second Edition of the National Nutrition Day carried out in 2019 was dedicated to food waste and was entitled “Nutrinformation: Waste on the Plate”^5^. Representatives from all the levels of the supply chain, from the primary sector to the distribution level, participated in this event, alongside academic researchers, representatives from government ministries, and students. It was an occasion to share knowledge and experience and demonstrate a high level of interest in the topic of FLW. To be as practical as possible and go beyond providing purely academic information, a catering school involved in food waste prevention projects was asked to prepare the lunch using recipes made from leftovers, giving a first-hand view of how to avoid waste.
- A chapter dedicated to FLW was included in the Italian Food-based Dietary Guidelines that were updated in 2018 by the CREA Food and Nutrition Research Center (28). The last chapter of the guidelines deals with sustainable diets and the food waste prevention issue is presented as one of the main strategies to pursue the sustainability goals.

**Figure 1:**
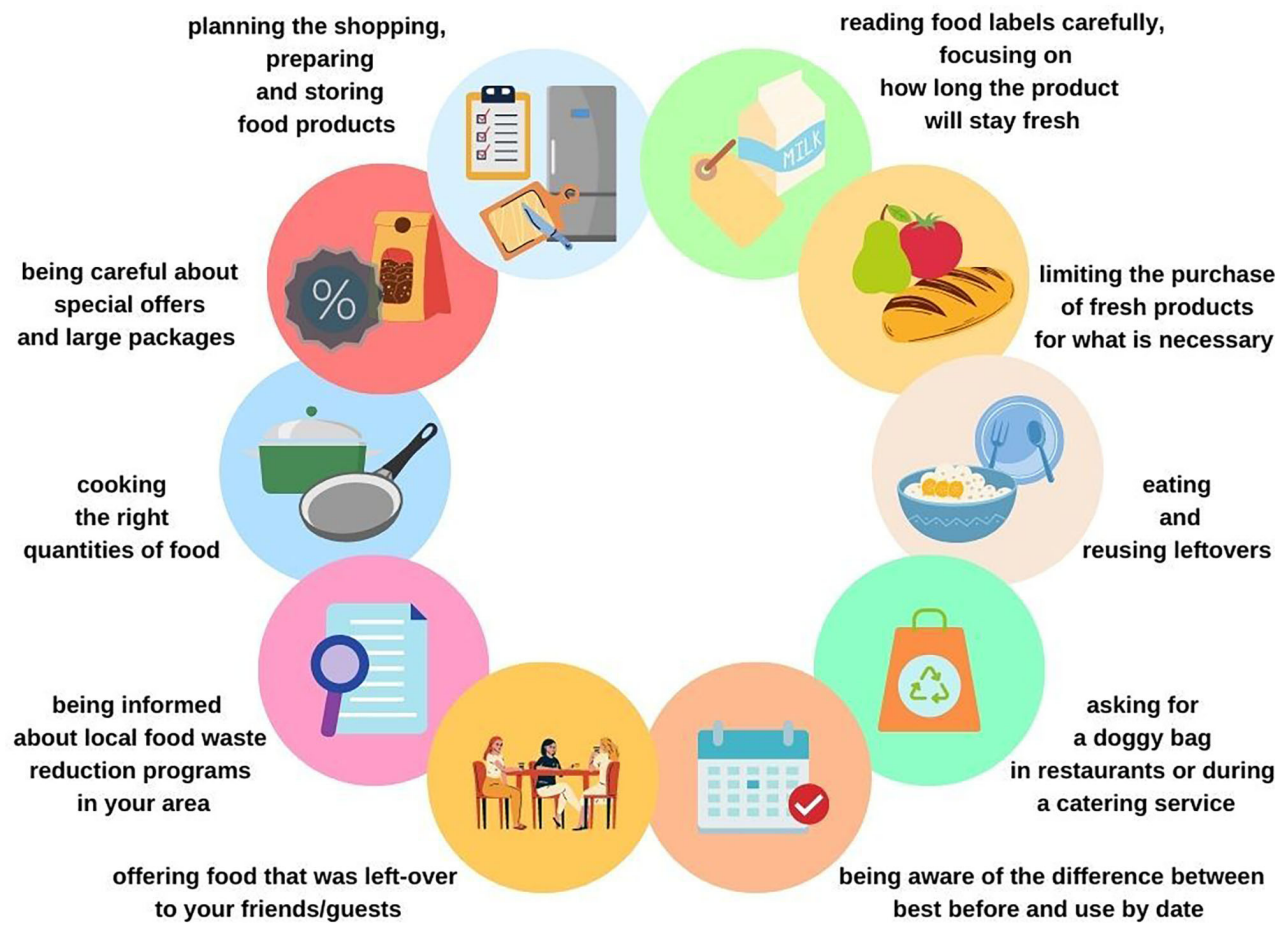
The 10 recommendations made by the OERSA to prevent food waste by consumers.

Several other communication strategies aimed at preventing FLW were put in place by the observatory. Among these is the “Decalogue against Food Waste,” a set of 10 recommendations (29) that can be publicized in schools, events, and information sessions. The 10 recommendations are shown in Figure 1.

Another educational tool produced as a food waste (FW) prevention actionable recommendation was a booklet with creative recipes (30) showing how to use no longer fresh foods that have been in the fridge for a while as well as how to prepare meals with leftovers.

In Italy, in addition to the OERSA, other actors are actively involved in developing strategies against food waste.

- The Waste Watcher International Observatory (31) was setup in 2013 as a spin-off of the University of Bologna. The Waste Watcher International Observatory provided data on household food waste and studied the behaviors of consumers in terms of food management. In the recent years, circular economy and sustainability issues have also been included in its activity. A national awareness campaign called “Zero Waste” involving experts, both in the private and public sectors, and citizens organized various activities with the aim of sharing best practices in the prevention of food waste and general waste, to reduce the waste of water and energy and optimize the use of land.
- The Food Sustainability Observatory (32) was setup in 2017 by the School of Management, Polytechnic University of Milan, and deals with the analysis and dissemination of best practices for innovation aimed at increasing the sustainability of production, focusing on the circular economy model in the agri-food system and on a more sustainable supply chain model. This observatory conducts research, awareness campaigns, and promotion activities for the food supply actors.

## Discussion

The policy framework described above shows that the OERSA is actively pursuing the directive objectives established during its development. The OERSA plays a pivotal role in Italian action related to FLW, as a technical entity is able to produce reliable data that can be used as a driver for policy actions. According to recent European estimates (33), consumers generate the highest amount of food waste. Educational programs could be one of the main strategies to tackle the problem and the recommendations developed by the OERSA are coherent with this. Increasing the awareness of consumers, who influence the entire supply chain production, can positively influence the management of the agri-food system, which, in turn, can reduce the generation of FLW. Regarding the primary sector—the second largest contributor to food waste after consumption level (33), the OERSA studies have given a positive contribution by adding information on this aspect. However, some areas have remained largely unexplored in terms of direct measurements. The data collected by the OERSA concerned only fruit, but research needs to be done also for vegetables, cereals crops, and animal products. For example, high discard rates are reported for fishing (10.1% of annual catches) called bycatches (34). In this case, although the donation process could run into difficulties related to the management of this kind of product, alternative destinations for the discard, such as the processing industry, could be identified.

The food supply actors pointed out that one of the main destinations of the surplus food recovered is that of donations for human consumption. This finding was supported by data collected by the Banco Alimentare Organization (35), highlighting that redistribution for human consumption is a strategy that is now consolidated across the food supply chain in Italy. The Banco Alimentare activity and similar non-profit initiatives that work on the Italian area are the main consequences of the incentives and subsidies established by Italian regulations, in particular by the Law 166/2016. However, even though this approach respects the food waste hierarchy order, there is always a variable amount of food that unavoidably becomes waste. In addition, the recovery process involves costs for the storage and the management of food surplus or in some cases also for processing it before it can be donated. The expense for food surplus management was estimated at between 0.05 and 0.1 €/kg for manufacturing companies, 0.4 and 0.8 €/kg for retail shops, and 1.5 and 2 €/kg for restaurants and canteens in Italy (36). As previously mentioned, the donation of food surpluses is an important aspect of the Italian normative framework and the OERSA was developed in view of this. However, considering the food waste hierarchy, the circular economy (CE) strategy action plan (37) may be an efficient solution to achieve sustainable production patterns (38, 39). For example, reusing food waste for animal feed (40, 41) maybe one of the more suitable options to insert a food product that could, otherwise, be thrown away back in the food supply chain.

Nevertheless, the food waste hierarchy stresses prevention as a priority strategy. In their systematic review, Redlingshöfer et al. (42) stated that there are many problems related to the adoption of actions aimed at the hierarchy of principles including that of prevention. The analysis pointed out that food waste prevention presents many obstacles such as insufficient public participation, perceived high costs, inadequate stakeholder engagement, and uncertainty in terms of policy performance. Additionally, models for improvement, examples, and best practices to create new policies in food waste prevention are insufficiently reported. The OERSA tried to overcome these criticisms and in consideration of the actions described in this article, it could be said that Italy is in a good position regarding the amount of data collected and the robust methodologies followed, which are then used as drivers to develop educational programs, awareness campaigns, and cooperation between the different sectors of the supply chain. In fact, although there are some aspects that need improvement, as a consequence of the commitment of the government to the FLW problem through the well-established legislation process starting with the Law 155 and ending with the Law 166, Italy has developed and implemented various policies (43) over the years, having increased the interest in FLW prevention and reduction.

Considering the European scenario, the OERSA recommendations are in line with the recommendations for action developed by the EU Platform on Food Losses and Food Waste (44). In fact, for the entire supply chain, the EU advocates cooperation between different actors of the agri-food system, an aspect strongly compatible with the nature of the OERSA, which originates from the FWPT and, therefore, includes representatives from the entire supply chain. At the consumer level, the EU focuses on establishing guidelines for the correct storage, management, and use of food and leftovers at home and for planning the shopping at the supermarket or at grocery stores. All these aspects are fully addressed by the OERSA communication campaigns and are compatible with the results of consumer surveys. For the primary sector, although the OERSA has not yet established specific guidelines, the dialog with the POs highlighted some points that also the EU recommends should be improved, i.e., a better alignment between supply and demand, allowing easy access to innovative strategies and strengthening financial support to farmers. A similar situation can be observed at the donation level. In this case, a common viewpoint can be seen regarding the innovation and modernization of the donation organization structure, together with the idea that donation is the best destination for food surplus.

Thanks to its work, the OERSA has obtained a comprehensive overview of the FLW phenomenon and has developed initiatives and strategies that have evolved in Italy over the years. Periodical meetings with the FWPT and with the actors that are tackling the FLW problem should be encouraged, following the EU recommendations regarding the creation of cooperation between different sectors. Concerning in particular the work of the OERSA, it is important that it is completed following the remaining directives, with the aim of carrying out studies on the distribution and foodservice sectors. The foodservice area needs particular attention considering that, due to the scarcity of data collected until now, it is impossible to obtain realistic estimates and an understanding of the causes of FLW in this sector. Obtaining this kind of information will provide instruments to improve the management of meals and to better design menus, which is of particular importance when referred to the school canteens (45–47). In fact, helping children to become aware of the consequences of food waste means educating future generations with appropriate sensitivity to this problem.

To conclude, the OERSA initiatives for tackling the FLW problem could represent a cornerstone and a valuable experience strictly connected not only to the environment, but also with diet and nutrition. The OERSA will continue its work looking for innovative proposals aimed at making production more efficient, in terms of a reduction in food losses or better management of food surplus, to reduce the environmental impact of the supply chain. The work on food waste at the household level and, in general, at the consumer level is inextricably linked to food consumption, food behavior, and other dietary aspects linked to nutrition and to the promotion of a varied, healthy, and sustainable diet.

## Author Contributions

FG and LR equally contributed to conceiving, writing, and reviewing the manuscript. LR was responsible for overall supervision, project administration, and funding acquisition. Both authors have read and agreed with the published version of the manuscript.

## Funding

This study was supported by the Italian Ministry of Agricultural, Food, and Forestry Policies under Grant 1456/2018 Annual Program against food waste. The funder had no role in the design of the study, in the collection, analyses, or interpretation of data, in the writing of the manuscript, or in the decision to publish the results.

## Conflict of Interest

The authors declare that the research was conducted in the absence of any commercial or financial relationships that could be construed as a potential conflict of interest.

## Publisher's Note

All claims expressed in this article are solely those of the authors and do not necessarily represent those of their affiliated organizations, or those of the publisher, the editors and the reviewers. Any product that may be evaluated in this article, or claim that may be made by its manufacturer, is not guaranteed or endorsed by the publisher.

## Footnotes

^1^ https://www.crea.gov.it/web/alimenti-e-nutrizione/-/osservatorio-sugli-sprechi-alimentari

^2^ https://www.facebook.com/OERSA-103469301798441/

^3^ https://www.instagram.com/oersa_ita/

^4^ https://twitter.com/_OERSA

^5^ https://www.crea.gov.it/-/nutrinformarsi-lo-spreco-nel-piatto
